# Current views on mechanisms of the FLASH effect in cancer radiotherapy

**DOI:** 10.1093/nsr/nwae350

**Published:** 2024-09-30

**Authors:** Yuqi Ma, Wenkang Zhang, Ziming Zhao, Jianfeng Lv, Junyi Chen, Xueqin Yan, XiaoJi Lin, Junlong Zhang, Bingwu Wang, Song Gao, Jie Xiao, Gen Yang

**Affiliations:** State Key Laboratory of Nuclear Physics and Technology, School of Physics, Peking University, Beijing 100871, China; State Key Laboratory of Nuclear Physics and Technology, School of Physics, Peking University, Beijing 100871, China; State Key Laboratory of Nuclear Physics and Technology, School of Physics, Peking University, Beijing 100871, China; State Key Laboratory of Nuclear Physics and Technology, School of Physics, Peking University, Beijing 100871, China; State Key Laboratory of Nuclear Physics and Technology, School of Physics, Peking University, Beijing 100871, China; State Key Laboratory of Nuclear Physics and Technology, School of Physics, Peking University, Beijing 100871, China; Oncology Discipline Group, the Second Affiliated Hospital of Wenzhou Medical University, Wenzhou 325003, China; Beijing National Laboratory of Molecular Science, College of Chemistry and Molecular Engineering, Peking University, Beijing 100871, China; Beijing National Laboratory of Molecular Science, College of Chemistry and Molecular Engineering, Peking University, Beijing 100871, China; Beijing National Laboratory of Molecular Science, College of Chemistry and Molecular Engineering, Peking University, Beijing 100871, China; Guangdong Basic Research Center of Excellence for Functional Molecular Engineering, School of Chemistry, Sun Yat-sen University, Guangzhou 510275, China; KIRI Precision Particle Therapy Flash Technologies Research Center, Guangzhou 510700, China; State Key Laboratory of Nuclear Physics and Technology, School of Physics, Peking University, Beijing 100871, China

**Keywords:** ultra-high dose-rate irradiation, FLASH effect, radiotherapy, mechanism

## Abstract

FLASH radiotherapy (FLASH-RT) is a new modality of radiotherapy that delivers doses with ultra-high dose rates. The FLASH effect was defined as the ability of FLASH-RT to suppress tumor growth while sparing normal tissues. Although the FLASH effect has been proven to be valid in various models by different modalities of irradiation and clinical trials of FLASH-RT have achieved promising initial success, the exact underlying mechanism is still unclear. This article summarizes mainstream hypotheses of the FLASH effect at physicochemical and biological levels, including oxygen depletion and free radical reactions, nuclear and mitochondria damage, as well as immune response. These hypotheses contribute reasonable explanations to the FLASH effect and are interconnected according to the chronological order of the organism's response to ionizing radiation. By collating the existing consensus, evidence and hypotheses, this article provides a comprehensive overview of potential mechanisms of the FLASH effect and practical guidance for future investigation in the field of FLASH-RT.

## INTRODUCTION

Cancer is one of the leading causes of human mortality at present [1]. Nowadays, ∼50% of cancer patients need radiotherapy as a primary or adjunctive therapy [2]. The goal of improving radiotherapy efficacy is to effectively kill tumor tissue while minimizing the damage to peripheral healthy tissue. FLASH radiotherapy (FLASH-RT) with an ultra-high dose rate (UHDR, typically ≥40 Gy/s) is an emerging technique to achieve this goal [3,4].

**Figure 1.**
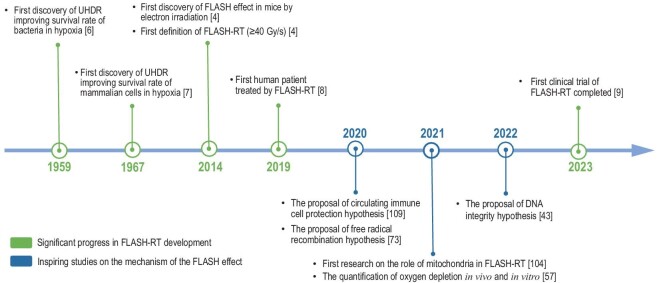
Big events in the development of FLASH-RT.

Exploration into the biological effects of UHDR radiation dates back to the last century. In 1958, Kirby-Smith and Dolphin first demonstrated the decreased chromosomal aberrations in *Tradescantia* microspores and pollen at high dose rates (1 and 4.0 × 10^6^ Gy/s) of irradiation compared with low dose rates (≤0.01 Gy/s) of irradiation [5]. In 1959, Dewey and Boag found that, under hypoxia conditions, the survival fraction of bacteria (*Serratia marcescens*) exposed to UHDR irradiation was higher than that of the conventional dose-rate irradiation [6]. It was the first report to show that FLASH irradiation (FLASH-IR) has a protective effect on living organisms. Later, this protective effect of FLASH-IR was demonstrated in mammalian cells [7]. In 2014, a groundbreaking *in vivo* study performed by Favaudon *et al.* revealed that, compared with conventional irradiation (CONV-IR, 0.03 Gy/s), FLASH-IR (>40 Gy/s) minimizes radiation-induced lung fibrosis in mice while retaining the toxicity to the lung tumor [4] and is now called the FLASH effect. This work launched an upsurge in physicochemical and biological research into the field of FLASH-RT. Numerous studies on the FLASH effect have been conducted, encompassing the exploration of its mechanisms and clinical trials (Fig. 1).

Clinical trials of FLASH-RT have achieved promising initial success [8,9]. In 2019, a pioneer study compared electron FLASH-RT and CONV-RT in a 75-year-old patient with recurrent cutaneous T-cell lymphoma on both elbows [8]. Researchers evaluated acute reactions and monitored the patient for 2 years, reporting comparable efficacy in tumor control and adverse effects between the two radiation modalities [10]. From 2020 to 2022, Cincinnati Children's Proton Therapy Center applied proton FLASH-RT to patients with extremity bone metastases as palliative treatment (FAST-01), which demonstrated comparable adverse events to standard treatment methods [9]. The preliminary clinical trials support the acceptable safety profile and feasibility of FLASH-RT as a treatment modality. Ongoing clinical trials are now underway to investigate a broader range of carcinomas, including thoracic bone metastases at Cincinnati Children's Hospital (FAST-02) [11], basal and squamous cell carcinomas at Lausanne University [12], etc.

**Figure 2.**
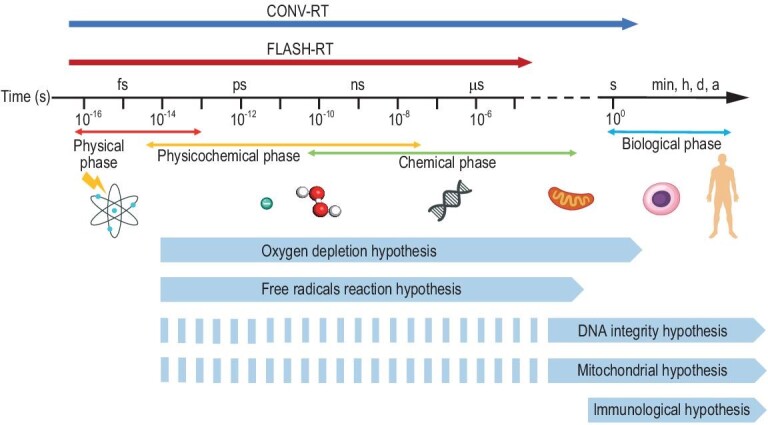
Schematic diagram of the radiation effect at different timescales. The timescales of mainstream hypotheses are shown (the dotted line means not yet proven by experiment or simulation).

However, several issues may hinder the clinical transition of FLASH-RT [13,14]. Firstly, the assessment of whether cancer patients are suitable for FLASH-RT is still inadequate, with a lack of preclinical and clinical trial data across various cancer types. Secondly, the available radiotherapy devices for FLASH-RT implementation are not popularized nowadays, necessitating an upgrade to currently available devices or the exploration of innovative accelerators [15,16]. Thirdly, the optimal parameters of FLASH-RT such as total dose, dose rate and radiotherapy fractionation are still unknown and have yet to be determined. Lastly and most importantly, the exact mechanism of the FLASH effect is unclear, which poses a significant obstacle to the development of FLASH-RT. The clinical application of FLASH-RT requires a better comprehension of the physicochemical and biological mechanisms of the FLASH effect. The demand for experimental evidence on different timescales (Fig. 2) also puts forward more diverse new requirements for the setting of experimental conditions.

Based on the ionizing radiation source, UHDR delivery methods employed in FLASH-RT experiments can be classified into three types: electron, proton (as well as heavy ion) and photon (X-ray). FLASH-IR experiments with electron irradiation were performed in the early years [5,7] and the discovery of the FLASH effect [4] stimulated a great number of animal experiments with electron FLASH-IR. The sparing effect of electron FLASH-IR has been demonstrated on a variety of normal tissues [17–23]. Meanwhile, experiments have shown the equivalent tumor control efficacy of electron FLASH-IR compared with CONV-IR in various tumor models [4,24,25]. Electron irradiation is also the first type used for FLASH-RT on a human patient [8].

Compared with electron FLASH, the development of proton FLASH is relatively late. It is noted that, as the proton beam has the characteristic of Bragg Peak in tissues, the combination of FLASH-IR and proton irradiation may better benefit normal tissue protection. Since the first demonstration of the FLASH effect by using proton irradiation [26], proton FLASH has been proven to be effective in various models [26–32]. Besides, some studies confirmed the effectiveness of proton FLASH-IR by utilizing Spread-Out Bragg Peak (SOBP) [27–29]. On the other hand, many experiments unveiled equivalent [32,33] or even improved [34,35] tumor suppression by using proton FLASH-IR compared with CONV-IR in different tumor models. Cincinnati Children's Proton Therapy Center conducted the first clinical trial of proton FLASH on 10 patients with bone metastases (FAST-01) [9]. As for heavy ion irradiation, Tinganelli *et al.* performed *in vitro* and *in vivo* FLASH studies with carbon ions [36,37] and revealed the induction of the FLASH effect by using carbon. Besides, helium-ion FLASH-IR was also shown to be valid in non-tumor-cell sparing under hypoxic conditions [38] and in sparing the body development of zebrafish embryos compared with CONV-RT [39]. Since heavy ion irradiation has high linear energy transfer (LET) and mainly causes direct damage, the verified FLASH effect by these heavy ions may be beyond the interpretation of oxygen-related hypothesis.

X-ray is the most commonly used type of radiation in clinical radiotherapy and Montay-Gruel *et al*. [40] first demonstrated the X-ray-triggered FLASH effect in mouse brain by using the kilovoltage (kV) X-rays generated at European Synchrotron Research Facilities. However, kV X-rays are considered unfeasible for treating deep-seated tumors because of the rapid decrease in the mean dose rate with the penetration depth in tissues [41]. Therefore, megavoltage (MV) X-rays were further developed for the research of FLASH-RT. Based on the MV X-ray generated by bremsstrahlung electrons, researchers delved deeper into the biological effect of X-ray FLASH-RT [42–44].

Nevertheless, whether it is electron FLASH [45,46], proton FLASH [21,47] or X-ray FLASH [48], negative experimental results against the FLASH effect have been observed both *in vitro* and *in vivo*, which demonstrates that the average/instantaneous dose rate is not the only decisive factor in the FLASH effect [49]. The complex and unclear condition of the FLASH effect underscores the importance of exploring the specific mechanism behind it, which will pave the way for the future clinical application of FLASH-RT. A convincing hypothetical mechanism should answer two questions: how does the dose rate affect the radiotherapy efficacy differently between FLASH-RT and CONV-RT, and what inherent differences lead to the opposite responses in normal and tumor cells? This paper introduces several mainstream explanations of the mechanism of the FLASH effect, including the oxygen depletion hypothesis, free radical reaction hypothesis, DNA integrity hypothesis, mitochondrial hypothesis, immunological hypothesis and other possible mechanisms. These hypotheses contribute reasonable explanations for the FLASH effect but on different spatio-temporal scales (Fig. 2).

## POSSIBLE MECHANISMS OF THE FLASH EFFECT

### Oxygen depletion hypothesis

The mechanism of oxygen depletion has gained the most popularity among various possible mechanisms. Early during the 1980s, scientists reached a consensus regarding the protective role of low oxygen concentrations during low-dose-rate irradiation. The difference in biological lethality between normoxic and hypoxic conditions is known as the oxygen enhancement ratio (OER) [50]. As for FLASH-IR, Dewey and Boag first illustrated the correlation between increasing dose rates and oxygen depletion by comparing the survival curves of bacteria that were irradiated at UHDR and conventional dose rates under hypoxic conditions [6]. Earlier experiments have shown the relationship between hypoxia and the protective effect of FLASH-IR [7,51]. Montay-Gruel *et al.* [17] demonstrated that the oxygen concentration is a key factor in the FLASH effect for the first time in animal experiments. Adrian *et al.* [24] also reported that hypoxia within cancer cells (prostate cancer cell line DU145) contributes to the radio-resistance to FLASH-IR. A recent experiment conducted by Leavitt *et al.* [52] reported the same antitumor efficacy of FLASH-IR under acute hypoxia. Also, in addition to normal tissue sparing, FLASH-IR could overcome hypoxia-mediated tumor resistance. The influence of hypoxia and oxygen concentration on the effectiveness of FLASH-RT has long been a focal point of scientific investigation.

Based on these experimental phenomena, researchers hypothesized that the sparing effect of FLASH-RT may result from the rapid depletion of oxygen [53]. Ionizing radiation causes indirect damage by generating free radicals through water radiolysis and the following reactions, especially in low-LET types of radiation (e.g. X-ray) [54]. By reaction with oxygen molecules, these radicals can form the oxygen peroxyl radical (DNA-OO•), which is a more severe and irreparable state of damage [53]. As FLASH-IR consumes oxygen in a short time through the following two reactions: e^−^_solv_ + O_2_→O_2_^−^•, H• + O_2_→HO_2_•, it may produce a significant reduction in oxygen concentration, thereby preserving DNA from transitioning into an irreversibly damaged state [55]. Kusumoto *et al.* evaluated the radiation chemical yield (G values) of 7-hydroxy-coumarin-3-carboxylic acid under proton irradiation, which can be generated through an oxygen-consuming reaction under radiation. They reported a significant decrease in production under an equal dose but greater dose rate and verified an instantaneous hypoxia under FLASH-IR [56]. On the other hand, CONV-IR delivers doses in a timescale of minutes, which is much longer than the reoxygenation time in cells, resulting in only a minor reduction in oxygen concentration [57]. The unchanged tumor-killing efficacy may be explained by the relatively hypoxic environment in tumors, where the reduction in cell oxygenation after FLASH-IR is comparatively minimal, leading to inadequate changes in radiosensitivity [3].

The essence of validating the oxygen depletion hypothesis is to clarify how scarce oxygen should be in order to manifest significant protection in normal cells against tumor cells and to estimate that value in clinical practice. Therefore, real-time monitoring of oxygen concentrations is imperative, which should ideally have a temporal resolution in milliseconds due to the nature of the rapid oxygen depletion during FLASH-IR [58]. The quenching of fluorescent or phosphorescent dyes by using oxygen is a common technique for measuring oxygen consumption during FLASH-IR [59]. Cao *et al.* [57] measured and compared oxygen consumption using the Oxyphor 2P probe under electron FLASH-IR and CONV-IR. *In vitro* experiments [57] showed comparable oxygen depletion under FLASH-IR (0.16–0.17 mmHg/Gy) and CONV-IR (0.19–0.21 mmHg/Gy), which is consistent with previous experimental results [58]. As for *in vivo* experiments [57], the total decrease in oxygen after a single fraction of 20 Gy of FLASH-IR was 2.3 ± 0.3 mmHg in normal tissue and 1.0 ± 0.2 mmHg in tumor tissue, whereas no decrease in oxygen was observed in the CONV-IR mode. This study demonstrated that FLASH-IR does deplete oxygen in tissues compared with CONV-IR, but not enough to induce sufficient hypoxia and a consequent significant reduction in radiosensitivity, since the physiological oxygen level in tissues is ∼38 mmHg [60]. However, it should be noted that, in the experiment performed by Cao *et al.*, the temporal resolution of measurement (∼150 ms) was much larger than the reaction time of FLASH-IR (∼74 ms in the research) [57]. Oxygen recovery and interactions during this period cannot be measured and remain partially unclear, which suggests that the conclusion may underestimate the extent of depletion.

Compared with experimental measurements, simulations offer a more detailed insight into the effects of reactions and parameters, aiding researchers in comprehending the process of oxygen depletion during irradiation. Boscolo *et al.* [61] did a Monte Carlo simulation by focusing on the chemical track evolution of 1-MeV electrons transmitting in water and the simulated oxygen consumption rate (∼0.33 μM/Gy or 0.19 mmHg/Gy) agreed with previously measured values [58]. To assess the effect of oxygen consumption on cell radiosensitivity under FLASH-IR, researchers developed relevant models, some of which took into account oxygen diffusion and cell survival curves [55,62]. Current modeling studies indicate that the typical doses delivered in FLASH experiments (∼20 Gy [4,17,22]) are not sufficient to cause adequate oxygen depletion in normal tissues; only under extreme hypoxia may the effect of oxygen depletion become more pronounced. This corresponds with many *in vitro* studies which have reported that only under the condition of a relatively low oxygen fraction (<2% of volume, depending on experimental set-up and cell source) does a difference between CONV-IR and FLASH-IR exist [24,38].

Recent developments have brought forth several challenges to the oxygen depletion hypothesis. The protective effect of FLASH-IR on normal cells has been proven in *in vitro* experiments conducted under normoxia conditions [20,63,64], in which oxygen depletion appears to play a minimal role. In addition, since the OER of heavy ions is close to 1, it suggests that the outcomes of heavy ion (e.g. carbon ion [37]) FLASH experiments bear little correlation with oxygen concentration, and thus cannot be explained by oxygen consumption.

**Figure 3.**
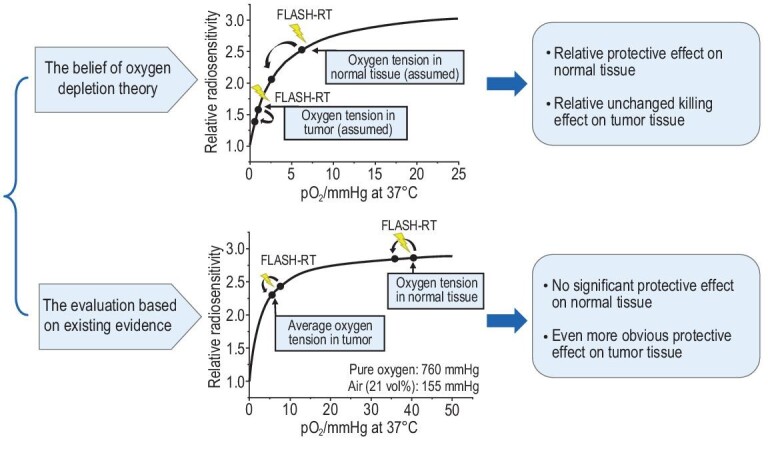
The illustration that oxygen depletion theory cannot fully explain the FLASH effect.

Further, the relationship between radiosensitivity and oxygen concentration is non-linear, as the physioxia point lies at the low slope of the oxygen concentration–relative radiosensitivity curve while the hypoxia point lies at the high slope [60]. The upper portion of Fig. 3 shows one qualitative explanation for oxygen depletion (adapted from the schematic diagram presented by Wilson *et al.*[3]) in which the radiosensitivity of normal tissue decreases more than that of tumor tissue after FLASH-IR. However, we quantified existing quantification results (∼40 and ∼10 mmHg originally, with 2 and 1 mmHg of depletion, for normal and tumor cells, respectively) [57,61] with the relationship between radiosensitivity and oxygen concentration [60] on the inferior portion of Fig. 3. It suggests that FLASH-IR cannot cause sufficient oxygen depletion in normal tissue. Further, the radio-resistance variation in tumor tissue appears to be even more pronounced, as the oxygen concentration of normal tissue is situated in the insensitive platform. Taken together, the experimental and simulation results contradict the hypoxia hypothesis strongly.

The aforementioned evidence from both theoretical and experimental studies casts doubt on the rationality of the oxygen depletion hypothesis. The quantification of concentration depletion is essential when discussing the oxygen depletion hypothesis, as the level of hypoxia affects the FLASH effect. However, quantitative data from neither experiments nor simulations show a considerable variation in radio-resistance that supports the oxygen depletion theory. Therefore, the FLASH effect can only be partially attributed to the oxygen depletion mechanism.

### Free radicals reaction hypothesis

Due to the limitations of the oxygen depletion hypothesis in explaining phenomena such as the FLASH effect on tissue cells in oxygen-rich environments [4], changes in radiosensitivity caused by oxygen concentration variation in cells may not be the primary factor that contributes to the FLASH effect. Instead, the impact of radiation on the chemical composition within cells appears to be more intricate and diverse [65]. Previous research has indicated that the irradiation of water can lead to water radiolysis and following chemical reactions [66]. This provides another avenue for investigating the FLASH effect.

**Figure 4.**
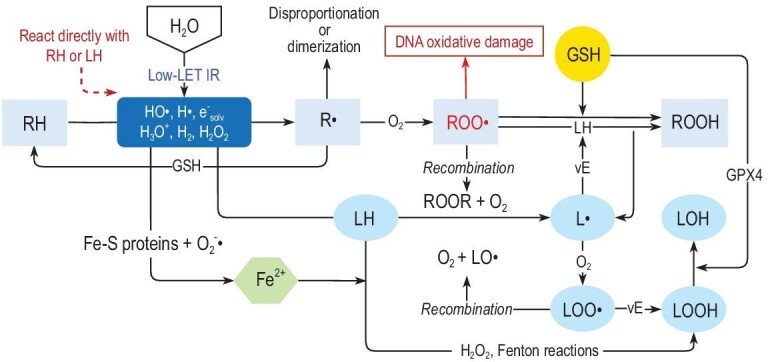
Illustration of free radical reaction caused by irradiation. The products of water radiolysis interact with biomolecules to produce organic free radicals, leading to oxidative damage by organic peroxyl radicals. Hydrogen peroxide may initiate ferroptosis through the Fenton reaction with unstable iron in cells and GSH plays a role in inhibiting ferroptosis. Recombination of radicals or interaction between peroxyl radicals and antioxidants (such as GSH, vitamin E, etc.) can terminate the reaction chain.

Compared with high-LET rays that cause damage directly, low-LET rays mainly induce cellular damage through indirect radical reactions. Radiation-induced indirect damage primarily refers to water radiolysis, which generates hydrated electrons (e^−^_solv_) and free radicals such as H• and OH•. Specifically, the free radicals that are generated from water radiolysis further react with biological molecules (RH) and with oxygen yield organic peroxyl radicals ROO• (Fig. 4), which are widely regarded as significant contributors to cellular damage. Besides, the radiosensitivity that is associated with oxygen is ultimately believed to be attributed to the action of ROO• [67]. It is worth noting that the e^−^_solv_ that is generated from the interaction in water can react with oxygen to produce O_2_^−^•. By interacting with iron-containing proteins, this species releases unstable active iron (Fe^2+^). Fe^2+^ can form a complex with oxygen, known as Fe^2+^–O_2_, which amplifies oxidative damage through the Fenton reaction [68].

Spitz *et al.* [69] initially explain the different cellular responses to FLASH-RT from the perspective of redox reactions. The key viewpoint is that, while the reaction of iron with O_2_^−^• generally comprises only ∼1% of other free radical reactions, the concentration of unstable iron in tumor cells is two to four times higher than that in normal cells [70]. This difference leads to increased damage in tumor cells during FLASH-RT compared with that in normal cells, which can promptly clear Fe^2+^ and ROOH. However, while the theory of Spitz *et al.* elucidates the differences between normal tissue cells and cancer cells under FLASH-RT, it does not account for the protective effect of FLASH-RT on normal tissue cells compared with CONV-RT. Furthermore, there are also some controversies regarding the discussion on the levels of hydrogen peroxide that are produced by cells under CONV-RT and FLASH-RT in their paper [71].

ROO•, as mentioned earlier, is considered a significant factor in causing cellular damage. On the one hand, it interacts with DNA, inducing chromosomal breaks, aneuploidy, mutations and thus cell death, while it also reacts with unsaturated lipids to generate ROOH, resulting in oxidative damage [72]. On the other hand, R• and ROO• can undergo self-recombination reactions, terminating the aforementioned reaction chain (Fig. 4). Building upon redox reactions, Labarbe *et al.* innovatively proposed the free radical recombination hypothesis for the FLASH effect [73]. In this framework, recombination reactions and reactions that cause cellular damage are considered to be competitive reactions. The FLASH effect can be explained by UHDR irradiation leading to rapidly elevated concentrations of R• and ROO•, which increase the proportion of recombination reactions and subsequently reduce cell damage. Labarbe *et al.* employed a large number of free radical reaction equations to construct a system of ordinary differential equations to mathematically simulate this hypothesis and quantitatively describe the damage with AUC(ROO•). Although results that were consistent with the FLASH effect were observed, the hypothesis neglected to explain the differences under FLASH-RT between normal cells and cancer cells.

For the interactions of free radicals, the role of antioxidants in these reactions has not received much attention. However, the balance between antioxidants and free radicals may play a role in determining the outcomes of oxidative stress. Hu *et al.* [74] expanded upon the free radical recombination hypothesis and emphasized the significance of antioxidants, thereby explaining the differences between normal tissue cells and cancer cells under FLASH-RT. ROO• reacts with a series of antioxidants (such as glutathione (GSH) and vitamin E, which is the major lipid-soluble antioxidant [75]) to produce ROOH [76] (Fig. 4). It is evident that the reaction between peroxyl radicals and antioxidants competes with the peroxyl radical recombination reaction, providing a possible explanation for the differences under FLASH-RT between cancer cells and normal tissue cells. Previous experiments have demonstrated that the proportion of the antioxidant GSH in cancer cells is higher than that in normal tissue cells [77]. On the one hand, a higher concentration of antioxidants that react with R• will result in the formation of fewer ROO•. On the other, the reaction between antioxidants and ROO• can inhibit the recombination of free radicals. This antioxidant-driven suppression of radical accumulation in cancer cells reduces the differences in free radical recombination between FLASH-RT and CONV-RT, making cancer cells more susceptible to the cytotoxic effects of FLASH-RT. In contrast, normal tissue cells, which have lower antioxidant reserves, may experience a greater disruption in the chain reactions of oxidative damage in free radical recombination under FLASH-RT compared with CONV-RT. This disparity leads to a protective effect in normal tissues, as fewer reactive oxygen species (ROS) are produced due to the rapid neutralization of peroxyl radicals by antioxidants. Consequently, FLASH-RT exhibits a cytotoxic effect on cancer cells while conferring protective benefits on normal tissue cells [74].

It is noteworthy that GSH has been shown to mitigate the oxidative stress that is induced by oxidants such as hydrogen peroxide. Spitz *et al*. [69] did not explicitly suggest that the process in their theory could potentially lead to ferroptosis, which is characterized by iron-dependent cell death that results from elevated levels of lipid peroxides that are catalysed by the Fenton reaction within cells. For lipids, LOOH with peroxidation can be reduced to lipid hydroperoxides under the action of GSH and GPX4, which is a GSH-dependent peroxidase. In systems in which ferrous ion (Fe²^+^) is released, ferroptosis can be induced and depletion of GSH may lead to inactivation of GPX4, thereby increasing sensitivity to ferroptosis [78]. GSH appears to suppress ferroptosis following irradiation, providing a protective effect. Furthermore, in cancer treatment, the antioxidant environment of the tumor is often considered to be one of the reasons for low radiation sensitivity, which is one of the problems that need to be overcome [24]. Conversely, in the context of the radical recombination and antioxidant hypothesis, GSH and the recombination reactions are mutually inhibitory. Tumor cells, which contain significantly higher levels of antioxidants compared with normal cells, exhibit less pronounced recombination reactions induced by ultra-high dose rates. From this perspective, GSH appears to play a protective inhibitory role. In these two processes, the role of GSH seems contradictory. Further research and exploration are needed to elucidate the relationship between them and the role of GSH in the FLASH effect.

The key reactions outlined in the aforementioned theories regarding ROS are illustrated in Fig. 4 [68,69,73,74,79]. While the potential chemical reactions that are induced by radiation are summarized here, this does not imply that all of them indeed play a role in the mechanism of the FLASH effect. Also, although the above-introduced theories seem sound, all the supporting evidence is mainly based on mathematical simulations. The work of Labarbe *et al.* has established a benchmark for the mathematical simulation of the free radicals reaction hypothesis [73]. The exceedingly short reaction window poses challenges in the experimental validation of these free radicals reaction theories. For example, the generation of free radicals such as HO• from water radiolysis occurs within the range of approximately 10^−12^ to 10^−6^ seconds after the initialization of irradiation [17]. In addition, due to the complex reaction network of biochemical molecules within cells post ionizing radiations, it is considerably challenging to comprehensively reflect the overall situation of biochemical reactions through the detection of specific components.

Recent advances have been made in understanding the reactions of free radicals in water under different dose rates [80,81]. By using scavengers for products such as hydroxyl radicals and hydrogen peroxide, researchers have investigated the differences in yields at high dose rates. The findings from existing studies are generally consistent, indicating lower yields of hydroxyl radicals and hydrogen peroxide under high dose rates. This result partly supports the notion that there are differences between FLASH-IR and CONV-IR during the chemical reaction stage. There have also been advancements in the study of lipid peroxidation. Portier *et al.* detected oxylipins under different irradiation conditions and found that, at 37°C, normal tissue cells exhibited lower levels of oxylipins under FLASH-IR compared with CONV-IR. This result was not observed at 20°C or in tumor cells [82]. Additionally, studies on lipid irradiation products, such as malondialdehyde (MDA), revealed that less MDA is produced under FLASH-IR [83]. These findings lend some support to the theory of lipid radical recombination. Although these studies are still some distance from fully validating the hypothesis of radical recombination, they provide a useful method for studying free radical reactions. It is crucial to identify scavengers that are highly specific to organic peroxy radicals. While studying the entire process may be challenging and real-time monitoring is currently not feasible, inferring the reaction process through the analysis of intermediate products remains a relatively convenient approach.

### DNA integrity hypothesis

As a result of ROS interaction and chromosome breakage, DNA fragments that leak and accumulate in the cytoplasm trigger the downstream inflammatory process. Through messenger cyclic GMP–AMP synthase (cGAS) and stimulator of interferon genes (STING) to the final product type I interferon (IFN-I), this cGAS–STING pathway recruits CD8^+^T cells to cause an immune response and inflammation. Therefore, DNA destruction evokes an immune response and may be an explanation for the differences in CONV-RT and FLASH-RT contexts.

Shi *et al.* [43] disposed the intestinal crypts of mice with 110–120 Gy/s of X-ray FLASH and observed a significant reduction in cytoplasmic double-stranded DNA (dsDNA) levels compared with 0.03 Gy/s of CONV-IR in mouse intestinal crypts. This reduction in nuclear-DNA fragment leakage partly suggests that FLASH-IR may alleviate DNA damage and inhibit the downstream cGAS–STING pathway. Based on this phenomenon, they proposed the DNA integrity hypothesis, suggesting that the rapid dose deposition of FLASH-IR (∼100 ms) minimizes the probability of DNA breakage, maintains genomic stability and reduces cGAS–STING signaling pathway activation.

Though they lack direct observational evidence, Shi *et al.*’s findings correspond to those from several recent *in vitro* experiments that involved the irradiation of DNA plasmids in water. Perstin *et al.* [84] used 16 MeV of electron FLASH-IR to irradiate DNA plasmids in water and found a significant reduction in both DNA single-strand breaks (SSBs) and double-strand breaks (DSBs). Two other experiments demonstrated that proton FLASH-IR reduced the induction of SSBs in DNA plasmids in water but did not significantly affect DSBs [85,86]. Immunofluorescence analysis of DNA damage response (DDR) proteins is a common method for DNA damage quantification, with γH2AX and 53BP1 widely used as markers for evaluating DSB damage foci [87]. It is essential to consider the timing of peak foci formation when evaluating the extent of irradiation-induced DSB damage, which is unnoticed by some research. After irradiation, cells rapidly recruit proteins such as 53BP1 to sites of DSBs, leading to a swift increase in the foci count. This count typically peaks within 15–30 minutes, during which only a minimal number of DSBs are repaired [88]. Therefore, the peak foci count can be approximated as representing the total number of radiation-induced DSBs. As DSBs are repaired, the foci gradually disappear and their numbers begin to decline, reflecting the ongoing repair process. Several studies have reported no significant difference in the induction of γH2AX/53BP1 foci at low doses between FLASH-IR and CONV-IR [43,64,89]. However, in the research of Buonanno *et al.* [64], γH2AX generation is reduced with FLASH-IR (1000 Gy/s) at higher doses (20 Gy). The observed variance in the foci count between FLASH-IR and CONV-IR may be attributed to FLASH-IR potentially generating clustered DSBs, as proposed by Atkinson *et al.* [90]. It can be inferred that FLASH-IR does not induce fewer DSBs compared with CONV-IR, but rather leads to a higher degree of DSB clustering with more DSBs per focus, resulting in fewer foci. At low doses, the extent of DSB clustering that is induced by FLASH-IR may not be significant enough to discern a notable difference in the foci count whereas, at higher doses, FLASH-IR may induce more pronounced DSB clustering, leading to a significant reduction in the foci count compared with CONV-IR. In addition, Kim *et al.* [25] revealed that FLASH-IR promotes expedited foci repair compared with CONV-IR. Since clustered DSBs have proven to be more beneficial for efficient repair [91], this phenomenon may be attributed to FLASH-IR inducing a higher degree of DSB clustering.

When characterizing DSB damage, the numbers of 53BP1 and γH2AX foci are generally similar or close. However, Fouillade *et al.* [20] observed fewer 53BP1 foci under electron FLASH-IR compared with CONV-IR, whereas the numbers of γH2AX foci were almost the same. This inconsistency may be due to the clustering of DSBs. 53BP1 exhibits liquid–liquid phase separation characteristics, which impose certain size limitations on its foci. In contrast, γH2AX, formed by the phosphorylation of histone H2AX, may have a less regular shape than 53BP1 (similar to spherical), which could lead to counting errors. This might explain the larger error bars in the γH2AX foci counts for MRC5 cells shown in fig. 1B of that paper [20].

Another widely used method for the assessment of DNA damage is the comet assay. By using the alkaline comet assay, Cooper *et al.* [92] proved that lower levels of DNA damage are induced following electron FLASH-IR—an effect that is modulated by the oxygen tension and increases with the total dose and dose rate of the irradiation. However, the difference in DSB induction may be missed in the study, which needs the validation of a neutral comet assay, since alkaline and neutral comet assays are sensitive for detecting SSBs and DSBs, respectively.

From the above discussion, the fact that FLASH-IR induces less SSB damage than CONV-IR may be concluded. This fact is coherent with the free radicals reaction hypothesis that was discussed in the previous section, as it is believed that radio-induced free radicals cause relatively non-lethal damage such as SSBs. At present, the research on the effect of FLASH-IR on DNA damage mainly focuses on foci quantification, while the quality of DNA damage and repair under FLASH-IR, such as the specific damage pattern and damage repair pathway, still requires further exploration.

Besides explaining how radio-resistance in normal cells can be enhanced by FLASH-IR, it is equally important to account for the same killing effect in tumor cells. This may be clarified by the unstable genome and weak DDR process of tumor cells [93], which prevents them from repairing conventional damage. Key signaling genes within the DDR pathway are often mutated post-cancerization, including PARP-1, which participates in DNA SSB repair [94]; ATM, which delivers ROS signals for radioresistant transitions; and ATR, which guarantees regular chromosome segregation through checkpoint responses [95]. Also, the relatively harmless SSBs for normal cells are likely to accumulate in tumor cells and finally convert into DSBs in the next DNA replication period. Genetic defects render tumor cells vulnerable and lead to considerable DNA fragment leakage under either CONV-IR or FLASH-IR, which stimulates inflammation through the downstream immunological pathway of IFN-I. Therefore, in the context of tumor cells, the distinction between SSBs and DSBs may be relatively less important, with dose emerging as the primary determinant of response, rather than the dose rate [96]. Nevertheless, a comprehensive understanding of the distinct responses in normal versus tumor cells under FLASH-IR requires further elucidation of the specific damage patterns and repair pathways that are engendered by FLASH-IR.

In addition to nuclear-DNA (nuDNA), mitochondrial DNA (mtDNA) may also play an important role in the FLASH effect, which will be discussed in the next section of this article.

### Mitochondrial hypothesis

Mitochondria play pivotal roles in a variety of cell-death pathways. First of all, mitochondria serve as the key organelles of cell endogenous apoptosis. Upon exposure to ionizing radiation, mitochondria generate excess ROS, leading to mitochondrial outer membrane permeabilization (MOMP) that is mediated by pro-apoptotic Bcl-2 proteins BAX and BAK [97]. Consequently, mitochondrial proteins such as cytochrome c (cyt c) are released into the cytoplasm, where cyt c binds with APAF-1 to recruit caspase-9 to form apoptosome, inducing an apoptotic reaction [98]. Apoptosis is generally considered to be an active programmed process of autonomous cellular dismantling that avoids eliciting inflammation [99]. In addition to apoptosis, mitochondria are also involved in multiple pro-inflammatory response pathways, which are mainly associated with passive and accidental cell death. One noteworthy pathway is that MOMP can promote the release of mtDNA into cytoplasm, which evokes the cGAS–STING pathway and induces the release of IFN-Ⅰ, leading to downstream inflammatory responses [100]. It has been shown that apoptosis-related caspases inhibit the induction of IFN-Ⅰ by mtDNA [101], suggesting a role for caspase-mediated apoptosis in preventing dying cells from triggering a host immune response. This interplay between caspase-mediated apoptosis and IFN-Ⅰ-mediated inflammation has also been demonstrated in ionizing radiation studies, in which the blocking of caspase-mediated apoptosis results in the increased release of mtDNA, the enhancement of immunity [102] and the induction of abscopal responses to radiotherapy [103]. These findings offer insights into strategies for enhancing the antitumor immune response or attenuating the normal tissue inflammatory response that is caused by radiotherapy.

Because of the vital role that mitochondria play in regulating cell death and immune response, researchers are curious about their potential role in the FLASH effect. Han *et al.* [104] conducted pioneering research on this topic by employing FLASH-IR (>10^9^ Gy/s of ultrafast laser-generated particles) and CONV-IR (0.05 Gy/s of Co_60_ γ radiation) to irradiate normal (cyt c^+/+^) and cyt c-defective (cyt c^−/−^) mouse embryonic fibroblast cells. Compared with normal (cyt c^+/+^) cells, the proportion of late apoptosis and necrosis in cyt c-deficient (cyt c^−/−^) cells was significantly lower under FLASH-IR in both hypoxia and normoxia, which implied that the loss of mitochondrial function may increase the resistance of cells to FLASH-IR. Guo *et al.* [63] carried out a comprehensive study in which they irradiated normal lung fibroblasts (IMR90) with protons under the conditions of FLASH (100 Gy/s) or CONV (0.33 Gy/s) under ambient oxygen concentration (21%). Compared with CONV-IR, FLASH-IR improved cell survival and prevented mitochondria damage whereas, conversely, the cell viability and mitochondrial morphology of lung cancer cells (A549) was negatively affected by both FLASH-IR and CONV-IR. The examination of the fate of irradiated cells showed that FLASH-IR did lead to apoptosis and possibly autophagy, while CONV-IR mainly induced necrotic cell death. Based on these findings, Guo *et al.* proposed a possible mechanism by which CONV-IR induces the dephosphorylation of p-Drp1, leading to mitochondrial fission and eventual cell necrosis, while proton FLASH-IR can mitigate this process, thereby preserving normal cell mitochondrial function. However, neither Han *et al.*’s work [104] nor Guo *et al.*’s work [63] explains how mitochondria influence the different responses of cancer and normal cells to FLASH-IR.

**Figure 5.**
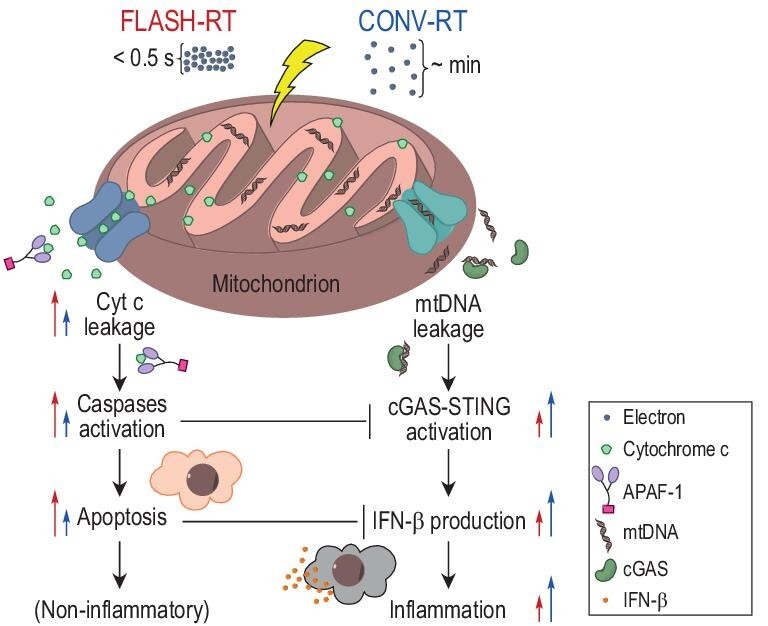
Illustration of the mitochondrial hypothesis [105]. FLASH-RT regulates the mitochondria-mediated apoptosis and inflammatory pathways to cause the FLASH effect.

To further explore the role of mitochondria in the FLASH effect, Lv *et al.* [105] conducted an inspiring study that unveiled the regulation of mitochondria-mediated apoptosis and inflammatory pathways by using FLASH-IR. They observed that, compared with low-dose-rate electron irradiation (0.36 Gy/s), FLASH-IR (61 or 610 Gy/s) amplifies cyt c leakage from mitochondria in MCF-10A human breast cells, which elicits substantial caspase activation but suppresses both cytosolic mtDNA accumulation and IFN-β production. This result indicates that FLASH-IR may enhance the programmed apoptosis that is mediated by the cyt c-caspases chain and inhibit the IFN-Ⅰ inflammatory response that is mediated by the mtDNA-induced cGAS–STING pathway, thus alleviating the immune damage to normal tissues post-irradiation (Fig. 5). In contrast, the cyt c leakage in MDA-MB-231 carcinoma cells after electron irradiation is limited, especially for the case of FLASH-IR, resulting in less cytosolic cyt c but stronger cGAS–STING activation than those in MCF-10A cells. Lv *et al.* [105] explained this difference as being due to the Warburg effect [106] that cancer cells tendentiously produce energy by aerobic glycolysis in cytoplasm rather than the oxidative phosphorylation and citric acid cycle that involves the participation of cyt c in mitochondria. Interestingly, in their research, the MCF-10A cells showed no significant change in mitochondrial morphology post-irradiation and even enhanced mitochondrial fission was observed in MDA-MB-231 cells post-FLASH-IR. Furthermore, the variation of cyt c leakage with increased irradiation dose rates was found to be inconsistent with the mitochondrial morphology change. These phenomena cannot be explained by the hypothesis about the Drp1 pathway that was proposed by Guo *et al.* [63]. Based on these findings, Lv *et al.* proposed the hypothesis of electron transport chain (ETC) disruption—that is, FLASH-RT stimulates extensive cascade feedback of ETC dysfunction and cyt c detachment from cardiolipin in normal cells, which is mediated by excessive ROS production and enhances the cyt c leakage from mitochondria to cytosol (supplementary fig. 5 of Lv *et al.*’s paper [105]). As for cancer cells, due to the cardiolipin and electron transport chain abnormalities [107], FLASH-RT could still disrupt the ETC function immediately but could not initialize substantial cyt c detachment nor then stimulate positive feedback. To prove the correctness of this hypothesis, the function of the ETC or the change in the mitochondrial membrane potential post-irradiation needs to be further investigated.

An alternative perspective on the potential function of mitochondria in the FLASH effect is from the perspective of mitochondrial ROS (mtROS) [108]. Researchers believe that FLASH-IR can reduce cell damage by limiting the increase in mtROS compared with CONV-IR in normal cells. As for tumor cells, the overproduction of mtROS renders them more susceptible an increase in mtROS levels. The specific mechanism can be referred to in the section on the free radicals reaction hypothesis.

### Immunological hypothesis

Previous sections have mentioned that radio-induced leakage of nuDNA, mtDNA and cyt c can activate downstream cell-death pathways and evoke an immune response, which is the typical pattern of a radio-induced immune response. Irradiation can influence the immune system in many ways, including affecting the circulating immune system, altering the composition of immune cells and immune molecules, inducing immunogenic cell death (ICD) and eliciting the abscopal effect. As the radio-induced immune response exhibits unique characteristics under FLASH-RT and plays a vital role in the FLASH effect, this section will discuss the potential immunological mechanisms of the FLASH effect. However, it should be noted that, as many studies have observed a difference between FLASH-RT and CONV-RT *in vitro* by using cell lines without co-culture with immunological cells [20,38,64,104], the immunological hypothesis alone cannot be regarded as a single comprehensive hypothesis to explain the FLASH effect.

Jin *et al.* [109] proposed the circulating immune cell protection hypothesis, highlighting the strong sparing effect of FLASH-RT on circulating immune cells through modeling and computation. They found that the number of lymphocytes that were affected by FLASH-RT in blood circulation is significantly lower than those affected by CONV-RT due to the relatively short irradiation time of FLASH-RT. Computation revealed a reduction in the killing rate of circulating immune cells from 90%–100% under CONV-RT to 5%–10% under FLASH-RT. The sparing effect of circulating immune cells diminishes the destruction of the body's immune system, helps the repair of damaged tissue cells and thus reduces the level of tissue damage. Cucinotta and Smirnova developed more comprehensive mathematical models to assess the level of surviving lymphocytes in the blood after FLASH-RT and their calculation results support the circulating immune cell protection theory [110–112]. The models take into consideration a series of parameters such as the dynamics of the lymphopoietic system in mice and the situations of two- or multiple-pulse irradiation. As for simulation, Galts and Hammi [113] exploited a dosimetric blood flow model to simulate the dose of circulating lymphocytes during CONV and FLASH pencil-beam scanning-based intensity-modulated proton therapy for brain tumors. The favorable simulation results verified the circulating immune cell protection by proton FLASH-RT during intracranial therapy.

To validate the computation and simulation results, experimental observations of the protective effect of FLASH-RT on circulating immune cells are imperative. Venkatesulu *et al.* [46] and Zhang *et al.* [47] reported negative results regarding the sparing effect of FLASH-RT on peripheral blood lymphocytes in mice by using 35 Gy/s of electron FLASH and 120 Gy/s of proton FLASH, respectively. However, no protective effects of FLASH-RT on normal tissues were observed in their experiments. Another study by Iturri *et al.* also found no difference in mice blood lymphocyte numbers between proton FLASH-RT and CONV-RT, but did demonstrate that FLASH-RT offers a neuro-protective effect and mounts an effective lymphoid immune response in the tumor [114]. These negative results challenge the circulating immune system protection hypothesis, suggesting that the effect of FLASH-RT on peripheral blood circulating immune cells may depend not only on the proportion of immune cells that are irradiated, but also on the complex damaging effects of UHDR irradiation, which abstract models with relatively invariant parameters may not capture. One possible explanation for the conflict result reported by Iturri *et al.* [114] is that the strong particle nature of proton irradiation makes proton irradiation with different dose rates have a similar killing effect on the flowing dispersed blood leukocytes. Under this condition, the total number of tracks may be more significant than the dose-rate effects. Conversely, for static tissues, proton irradiation can exhibit a dose-rate effect because the energy deposition of a single track matters here. Based on this explanation, experiments that use photon irradiation are recommended as a necessary comparison. In summary, the validity of the circulating immune cell protection hypothesis still needs further validation and exploration.

Apart from the circulating immune cell protection hypothesis, which explains the FLASH effect from the perspective of systemic immune responses, FLASH-RT has also been proven to influence immunity in local tissue. Specifically, FLASH-RT may alter the immune cell composition within the irradiated tumor microenvironment (TME) compared with CONV-RT, particularly regarding the recruitment of T lymphocytes, thereby affecting antitumor immunity. Rama *et al.* [34] conducted a FLASH experiment on a model in which Lewis Lung Carcinoma (LLC) cells were inoculated into the left lungs of mice. They observed increased recruitment of CD3^+^ T lymphocytes, particularly CD4^+^ and CD8^+^ cells, from peripheral tumor margins to the tumor core under FLASH-IR compared with CONV-IR. Based on a similar LLC mice model, Kim *et al.* [25] and Shukla *et al.* [35], respectively, proved that FLASH-IR increases the infiltration of cytotoxic CD8^+^ T lymphocytes within the tumor in comparison with CONV-IR, thus guaranteeing a better tumor-killing effect. Furthermore, in the research undertaken by Shukla *et al.* [35], FLASH-IR was more effective at decreasing the percentage of immunosuppressive regulatory T cells (Tregs) in T lymphocytes, decreasing pro-tumorigenic M2-like macrophages and increasing antitumor M1-like macrophages. The enhanced infiltration of lymphocytes in TME after FLASH-RT was also observed by Eggold *et al.* in the mice ovarian cancer model [115]. However, there are recent studies based on different tumor models that demonstrate no significant difference in T lymphocyte recruitment between FLASH-RT and CONV-RT [114,116,117], casting doubt on the previous discoveries. Consequently, while it is widely accepted that FLASH-RT can enhance T lymphocyte recruitment in TME similarly to CONV-RT, the potential for FLASH-RT to exert a more pronounced effect than CONV-RT remains to be further investigated. As for the immunological side effects of FLASH-RT on normal tissue, some studies have revealed the impact of FLASH-IR on the number of macrophages in neural tissue. They reported that, compared with CONV-IR, FLASH-IR reduced the activation of microglia (macrophages in nerve tissue), which in turn reduced the induction of neuroinflammation [23,29,118].

FLASH-RT may alter the expression of certain cytokines, thereby contributing to the FLASH effect. Transforming growth factor-β (TGF-β) is a multifunctional cytokine that plays an important role in regulating the immune system and tumor growth. The increase in TGF-β after exposure to ionizing radiation is proven to have side effects on different normal tissues, such as fibrosis induction [119]. Studies have shown that, compared with CONV-IR, FLASH-IR can reduce TGF-β production in normal tissues, including mouse lung [4], mouse skin [30,31], canine skin [31] and human lung fibroblasts [64]. This may explain the protective effect of FLASH-IR on normal tissues. In addition, TGF-β exhibits a dual role in tumor regulation. TGF-β plays a tumor-suppressive role in normal and early-stage cancer cells mainly by promoting apoptosis and inhibiting cell cycle progression, while it also plays a tumor-promoting role in late-stage cancer cells mainly by inducing proliferation, invasion, angiogenesis, metastasis and immune suppression [108,119]. Investigation of changes in TGF-β levels in tumor tissues after FLASH-IR could provide insights into its role in maintaining lethality in tumor cells. In addition to TGF-β, FLASH-IR was also proven to induce variations in other cytokines compared with CONV-IR, such as Cxcl-1, G-CSF, GM-CSF, IL-1β, IL-4, IL-6, IL-10, TNF-α and KC/GRO [30,44,118]. Various cytokines influence the immunological effects of FLASH-IR through a complex interaction network. The specific impact details need to be further investigated.

In recent years, a kind of unique clinical radiotherapy response—the abscopal effect—has attracted much attention; it refers to tumor regression at sites that are distant from the irradiated volume in radiotherapy [120]. The principle of the abscopal effect is thought to be related to ICD, mediated through two main mechanisms [121]. The first is the release of tumor associated antigen (TAA) and tumor specific antigen (TSA). The second is the release of specific damage-associated molecular patterns (DAMPs), such as calreticulin exposure on the surface of cell membranes, as well as the extracellular release of heat shock proteins, high mobility group protein B1 and adenosine triphosphate [122]. While there is substantial knowledge about ICD generation in CONV-RT, limited research has focused on ICD under FLASH-RT conditions and the subsequent abscopal effect. Shi *et al.* [43] demonstrated that, with the assistance of anti-PD-L1 therapy, X-ray FLASH-IR can produce the same inhibitory effect on both primary and secondary tumors as CONV-IR. This implies that FLASH-RT also has the potential to induce the abscopal effect. However, further verification of the abscopal effect under the condition of FLASH-RT necessitates experimental evaluation of various immune cells and immune molecules such as TAA, TSA and DAMPs involved in ICD.

### Other possible mechanisms

The effect of FLASH-IR on stem cells (both normal tissue stem cells and tumor stem cells) may also be an important part of the FLASH effect. Montay-Gruel *et al.* [18] first supposed that FLASH-RT might protect neural stem cells based on the known sparing effect of FLASH-IR on neural tissues. Later, Vozenin *et al.* [22] demonstrated the sparing effect of FLASH-IR on porcine skin epidermal stem cells. Stem cells typically reside in hypoxic niches that are distant from vasculature, leading to relative radio-resistance. Pratx *et al.* [55] stated that the FLASH effect might stem from the specific sparing of stem cells through modeling methods, as the typical oxygen tension in hypoxic stem cell niches overlaps with the range over which their model predicts significant radioprotection from oxygen depletion associated with FLASH-RT. Therefore, the sparing effect of FLASH-IR on normal tissues may be related to its protective influence on normal tissue stem cells. As for tumor stem cells, previous studies have highlighted their radio-resistance to CONV-IR [123]. Yang *et al.* [124] first demonstrated that tumor stem cells (MCF-7 cancer stem cells) were more radioresistant than non-stem tumor cells (MCF-7) under proton FLASH-IR. Their subsequent experiments that tracked lysosomes and autophagy revealed higher levels of lysosomes and autophagy in tumor stem cells. Therefore, the heightened radio tolerance of tumor stem cells may be associated with increased lysosomal-mediated autophagy as well as reduced apoptosis, necrosis and pyroptosis. The radioresistant characteristic of tumor stem cells provides clues for enhancing the FLASH effect and optimizing future clinical FLASH therapy.

Several studies have shown the potential of FLASH-IR to spare blood vessels. The first report on the FLASH effect revealed its ability to prevent acute apoptosis in blood vessels and bronchi in mice compared with CONV-IR [4]. FLASH-IR was found to outperform CONV-IR in protecting the integrity of brain microvessels, which may partly elucidate its role in preserving cognitive function [19,29]. The protective effect of FLASH-IR on blood vessels in normal tissues corresponds to its sparing effect on normal tissues. As for blood vessels in the TME, Kim *et al.* [25] found that, in the mouse model with LLC tumor transplantation, while CONV-IR induced a reversible collapse of blood vessels (detected 6 and 48 h post-irradiation), FLASH-IR did not cause a rapid or reversible collapse of blood vessels. In 2001, Jain introduced the concept of vascular normalization [125], indicating that the judicious use of antiangiogenic therapies does not block blood vessels, but rather reverts the severely abnormal structure and function of the tumor vasculature to a more normal state. Tumor vascular normalization enhances tumor perfusion, thus increasing oxygen and drug delivery to the tumor and augmenting the effect of radiotherapy and chemotherapy. From this point of view, the states of tumor blood vessels before and after irradiation, whether they exhibit normal or abnormal characteristics, will contribute to the antitumor efficacy. Interestingly, in the work performed by Kim *et al.* [25], there were no differences observed in the CD31-positive vessel number, caspase-3 levels and hypoxia levels between treatments with FLASH-IR and CONV-IR, indicating that the state of the tumor blood vessels remained unconverted. Further investigation into the effect of FLASH-IR on tumor blood vessels is needed.

## CONCLUSION AND PROSPECTS

This article discusses the possible mechanisms of the FLASH effect from the perspectives of oxygen depletion, free radicals, DNA damage, mitochondria, immunity and other possible mechanisms. Importantly, the mechanisms that were proposed above are not independent of each other, but are rather interconnected according to the chronological order of the response of the organism to ionizing radiation (Fig. 2). Upon exposure to ionizing radiation, the process of radiolysis and the interactions that follow produce free radicals, which will cause damage to DNA and result in dsDNA leakage into the cytoplasm. This indirect DNA damage can be enhanced by the presence of oxygen and is thus closely associated with the oxygen concentration. As for mitochondria, the radioactive process triggers the leakage of both mtDNA and cyt c. The presence of cytosolic dsDNA and cyt c can modulate the downstream immune responses via relevant signaling pathways, ultimately influencing the lethality of the radiation exposure. We may organize and elucidate the underlying mechanisms of the FLASH effect along this cascade of radiation responses. The instantaneous dose delivery of FLASH-RT prompts a rapid increase in the concentration of free radicals and diminishes the proportion of peroxyl radicals through free radical recombination. Consequently, this mitigates damage to biomolecules, including DNA, and reduces the leakage of dsDNA into the cytoplasm. Meanwhile, within mitochondria, the rapid escalation of free radicals during FLASH-RT promotes the dissociation and leakage of cyt c. The reduction in cytoplasmic dsDNA attenuates downstream inflammatory pathways, whereas the increase in cyt c enhances the apoptotic pathways that are related to programmed cell death, thus ultimately reducing the tissue toxicity from FLASH-RT. However, in the case of tumor tissues, distinctive characteristics such as different redox levels, DNA stability or the Warburg effect may lead to a breakdown in one of the aforementioned mechanisms, thereby preserving the unaltered response to FLASH-RT.

In recent years, with the deepening understanding of the FLASH effect, *in vitro* experiments that aim to study the mechanisms of FLASH effect have gradually sprung up. When *in vitro* experiments are performed, particular attention should be paid to the oxygen concentration—whether it is normoxia, physoxia, or hypoxia [49]. Different oxygen concentrations lead to different radiosensitivity, which may differ from the *in vivo* situations. In addition, it is crucial to note that *in vitro* experiments typically exclude the involvement of the immune system, which may impact cellular responses to irradiation and should be considered when analysing results.

Clinical trials of FLASH-RT that use both electrons and protons have achieved promising initial success [8,9]. However, the translation of FLASH-RT into clinical practice faces multiple challenges [13,14]. Primary concerns involve technical difficulties such as the upgrading and innovation of irradiation equipment and treatment planning systems, which should meet the specific requirements of UHDR. Determination of the optimal parameters for FLASH-RT is another pivotal issue. Apart from the exact dose-rate thresholds for triggering the FLASH effect, other physical parameters (such as total dose, irradiation time, beam energy and fractionation), radiation geometry and regimen, and biochemical and intrinsic biological conditions (such as oxygen tension, hypoxia, tumor and tissue type, and individual sensitivity to FLASH) should all be considered [13]. Additionally, logistical and patient-specific factors, such as eligibility criteria, safety assurance and follow-up management, also require careful planning and implementation.

The potential for clinical translation as well as the associated difficulties underscore the need for further exploration into the physicochemical and biological mechanisms of the FLASH effect. An in-depth understanding of these mechanisms will help to better identify the exact conditions that induce the FLASH effect and the optimization strategies to facilitate it. For example, studies that investigate the impact of oxygen concentration [52] and radio-induced immunity [35,114] will offer valuable insights for enhancing the efficacy of FLASH-RT. Simulation studies as well as *in vitro* radiochemistry research will also provide guidance on optimization of the dose rate/dose adjustments [73,113]. Therefore, with further exploration into the mechanisms of the FLASH effect, FLASH-RT holds significant promise for improving the therapeutic efficacy of radiotherapy.
